# Quantifying and mitigating electrical and environmental impacts of corona discharge

**DOI:** 10.1038/s41598-025-26521-z

**Published:** 2025-11-21

**Authors:** Ahmed M. Zobaa, Hazem H. Abdelnabi, Rodan M. Reda, Ahmed G. Mahmoud

**Affiliations:** 1 Electrical Power Engineering Department, Faculty of Engineering, Cairo University, Giza, Egypt; 2 Engineering Mathematics Department, Faculty of Engineering, Cairo University, Giza, Egypt

**Keywords:** Corona discharge, Electrical insulation, Environmental impacts, High-voltage transmission systems, Mitigation strategies, Power losses, Statistical analysis, Energy science and technology, Engineering, Mathematics and computing

## Abstract

Corona discharge has been recognized for centuries, with sailors reporting the bluish glow of St. Elmo’s fire on ship masts during storms. In the early development of high-voltage engineering, researchers such as Townsend and Peek described the physical basis of this phenomenon as the ionization of air around a conductor when the electric field exceeds the strength of the surrounding medium. The result is a partial discharge that produces visible light, hissing sounds, ozone, and other reactive gases, while also creating radio interference and ultraviolet radiation. In modern transmission systems, these effects appear as wasted power, accelerated wear of insulators, shortened equipment lifetime, and environmental concerns. Although corona has been studied for decades, it continues to challenge the reliable and economical operation of high-voltage networks, particularly under changing weather conditions. This study investigates the phenomenon by analyzing its causes, effects, and mitigation strategies through a combination of theoretical modelling, simulation, and statistical analysis. Using MATLAB Simulink and Python, simulations were conducted under varying environmental conditions—including temperature, humidity, and pressure—as well as electrical parameters such as voltage and conductor design, using observed data to ensure practical relevance. Comparable data sources may be used in other national or regional contexts. Key statistical techniques, including linear and multiple regression, analysis of variance (ANOVA), t-tests, and Monte Carlo simulations, were applied to determine the most influential factors affecting corona discharge losses. Results confirmed that higher voltage levels and unfavorable environmental conditions significantly increase corona loss, while increased conductor spacing and the use of corona rings emerged as the most effective mitigation strategies. An economic analysis based on probabilistic modelling estimated potential annual savings of up to 455 million Egyptian pounds (EGP) for the Egyptian grid, serving as a representative case study. The analytical framework is general and can be applied to other national transmission systems with appropriate data. The findings offer data-driven insights for improving transmission efficiency, minimizing power losses, and enhancing the overall reliability and cost-effectiveness of high-voltage power systems.

## Introduction

**Fig. 1:**
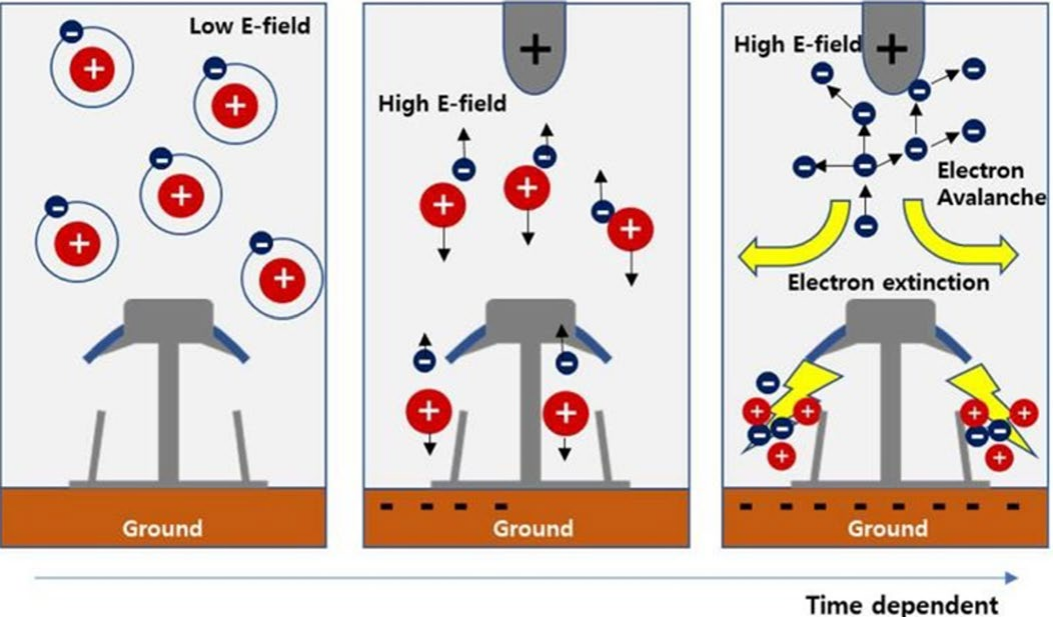
Schematic of molecular ionization by external high electric field.

 Electricity is essential for modern life, powering industries, transport, and communication. As high-voltage systems developed, engineers observed a hissing noise and light around transmission lines, which was later understood as corona discharge^1^. Corona discharge in high-voltage power systems has been extensively studied due to its detrimental impact on energy efficiency, electromagnetic interference, and infrastructure longevity. At its core, corona occurs when the electric field around a conductor exceeds the dielectric strength of air (≈ 30 $\:kV/cm$ at standard temperature and pressure (STP)), initiating ionization and a blue glow with audible noise Corona discharge causes power losses and harmful gas emissions, prompting extensive research^2^. Figure 1 illustrates the corona discharge initiation process under varying electric field strengths over time. In the first stage (left), under low electric field conditions, electrons remain bound to their parent atoms, and ionization is negligible. As the electric field increases (middle), the energy becomes sufficient to free electrons from atoms, creating positive ions and initiating movement toward the electrodes. In the final stage (right), a strong electric field leads to an electron avalanche, where free electrons accelerate and collide with neutral gas molecules, releasing more electrons in a chain reaction. This self-sustaining ionization process is characteristic of corona discharge^3^. The figure also depicts electron extinction zones, highlighting where recombination occurs or the ionization ceases due to field distortion or reduced carrier energy. This time-dependent mechanism is fundamental to understanding how corona discharge develops and why it becomes prominent near sharp conductor edges or under specific environmental conditions. The visual effectively complements theoretical models such as Peek’s Law by illustrating the microsale physics driving macroscopic power loss^4^.

Peek’s Law remains foundational, linking corona inception voltage (CIV) to conductor radius, spacing, and surface condition^5^. Recent research has also deepened understanding of streamer discharge phenomena under complex voltage conditions^6,7^. examined surface streamer characteristics under AC–DC composite voltages and strong vertical field components, respectively, providing critical insights into space-charge and streamer evolution. These findings align with corona discharge onset mechanisms described by Peek’s Law and reinforce the need for advanced discharge-aware insulation models. Moreover^8^, explored thermal fault detection in cable insulation using gas sensor arrays, highlighting the potential of sensor-based diagnostics to complement corona monitoring strategies. Environmental factors—humidity, temperature, pressure, and pollution—significantly modulate corona onset and loss magnitude. Absorption of water vapor and decreased air density exacerbate ionization, increasing power losses and ozone generation. Studies in Indonesia reported monthly corona power losses ranging from 0.4 kW to nearly 25 kW, with transmission efficiency falling to around 90%, driven largely by environmental variation^9^. Recent work has expanded beyond AC to include DC corona effects. Simulations and experimental modelling in wire–cylinder–plane geometries have provided deeper insight into DC-related ion distributions and space-charge dynamics^10^. Additionally, multi-electrode systems reveal complex self-synchronized Trichel pulse trains, emphasizing the need for refined space-charge-aware models in HVDC systems^11^. At the system design level^12^, proposed advanced reactive power control for hybrid DC transmission, addressing operational challenges closely related to corona-induced losses. Similarly^13^, developed dual-excitation permanent magnet linear machines with extended harmonic utilization, illustrating how machine design innovations intersect with transmission reliability and corona mitigation goals. Recent studies have emphasized not only the technical aspects of corona discharge mitigation but also the broader reliability and safety implications in large-scale power projects. For example^14^, examined risk pre-control in mega hydropower engineering, underscoring the importance of predictive diagnostics in complex electrical systems. Furthermore^15^, addressed reactive power control in hybrid DC transmission systems, which complements our analysis of corona discharge impacts on transmission efficiency and stability. Together, these works highlight the multidisciplinary significance of corona discharge research and motivate the need for comprehensive approaches that integrate electrical, environmental, and material perspectives. Mitigation strategies remain central: classical approaches include corona rings, grading rings, increased conductor diameter, and phase spacing—though their performance varies with environmental conditions. MATLAB-based studies extend Peek’s modelling to single- and multi-phase lines, confirming effectiveness of spacing, conductor radius, and voltage-control in eliminating corona under 400 kV^16^. Material innovations such as superhydrophobic/hydrophilic coatings and dielectric oil injection mechanisms offer novel mitigation pathways, e.g., coatings reducing corona currents 2–4×, and oil-based suppression systems in UHV lines^17^. Recent material-oriented studies also contribute to enhancing high-voltage insulation reliability. For example^15^, demonstrated advanced laminate designs with improved angular and electrical performance, highlighting the importance of structural damage tolerance under electrical stress. Similarly^12^, investigated bonding mechanisms in metal coatings, which can inform conductor and insulator surface treatments. At the nanoscale^18^, reported on heterostructure-based negative differential resistance devices, which reflect the growing potential of advanced materials for tailoring electrical behavior under high fields. An unexpected yet innovative area involves harnessing corona-generated energy for sensor power via HVDC transmission lines—demonstrating corona’s potential as a localized energy source. While not directly mitigating losses, such systems highlight opportunities to repurpose corona effects^19^.

Beyond material developments, accurate diagnostics and measurement are increasingly vital for corona-related studies^20^. modeled thermal responses in electro-explosive devices, underscoring the role of predictive simulations in evaluating electrical stress effects. Likewise^21^, used virtual measurement approaches to study wave propagation, offering methodologies applicable to high-voltage electromagnetic interference analysis^22^. further investigated electrothermal conduction in railway catenary systems, demonstrating how thermal-electrical coupling informs the design of insulation and corona mitigation technologies in real-world infrastructure.

This study aims to significantly attenuate corona discharge in high-voltage transmission systems in order to achieve multiple favorable outcomes. Specifically, it seeks to reduce maintenance and operational costs, minimize power losses to enhance overall system efficiency, and prevent long-term degradation of insulators caused by chemically active gases generated during discharge. To accomplish these objectives, the research integrates a comprehensive investigation of the physical, environmental, and design-related factors that influence corona behavior, with the ultimate goal of identifying and validating the most effective mitigation strategies.

Despite decades of research on corona discharge, it continues to present serious challenges in modern high-voltage transmission systems. The phenomenon leads to substantial power losses, electromagnetic interference, accelerated degradation of insulators, and the emission of harmful gases, all of which undermine transmission efficiency and environmental sustainability. While several mitigation strategies have been proposed—such as corona rings, conductor spacing adjustments, and material innovations—their effectiveness under varying environmental and electrical conditions remains insufficiently quantified. Moreover, the economic implications of corona-related losses are rarely assessed in an integrated framework. Therefore, there is a pressing need for a comprehensive study that simultaneously examines the physical causes, environmental influences, and financial impacts of corona discharge, while identifying and validating effective mitigation strategies for large-scale power systems. While this paper focuses on Egypt as a case study due to data availability and its alignment with vision 2030 energy efficiency goals, the proposed methodology and findings are generalizable and applicable to high-voltage transmission systems worldwide.

The advantages of the study can be summarized as follows:

- Provides a comprehensive framework that combines theoretical modelling, simulation, and statistical analysis for corona discharge evaluation.
- Quantifies the influence of environmental (temperature, humidity, pressure) and electrical (voltage, conductor spacing) factors on corona losses using real-world data.
- Validates the most effective mitigation strategies (corona rings and increased conductor spacing) through statistical testing.
- Incorporates an economic assessment, estimating potential annual savings of up to 455 million Egyptian Pounds (EGP) from targeted interventions.
- Aligns findings with national energy efficiency goals (e.g., Egypt’s 2030 Vision), ensuring direct relevance to policy and industry.
- Enhances the reliability, efficiency, and sustainability of high-voltage transmission systems by addressing power losses, interference, insulator degradation, and environmental impacts.

The main contributions of the paper can be summarized as follows:

- Comprehensive analysis of corona discharge: The study integrates theoretical modelling, simulations, and statistical techniques to investigate corona discharge phenomena in high-voltage transmission systems.
- Quantification of electrical and environmental impacts: Voltage, temperature, humidity, and pressure were statistically analyzed to determine their influence on corona loss, highlighting critical dependencies and thresholds.
- Validation of mitigation strategies: Design modifications such as the use of corona rings and increased conductor spacing were confirmed through analysis of variance (ANOVA) to significantly reduce corona-related power losses.
- Insulator design evaluation: Enhanced insulator materials demonstrated statistically significant improvements in long-term degradation resistance compared to standard designs.
- Economic impact estimation: A Monte Carlo simulation projected potential annual savings of up to 455 million EGP through the implementation of targeted mitigation strategies.
- Electromagnetic interference assessment: The study established that corona discharge disproportionately affects Amplitude Modulation (AM) radio bands, indicating a need for electromagnetic interference (EMI)-conscious infrastructure planning.
- Data-driven methodology: The combination of MATLAB Simulink modelling, Python-based statistical analysis, and real-world environmental data provides a robust framework for predictive diagnostics.

**Fig. 2:**
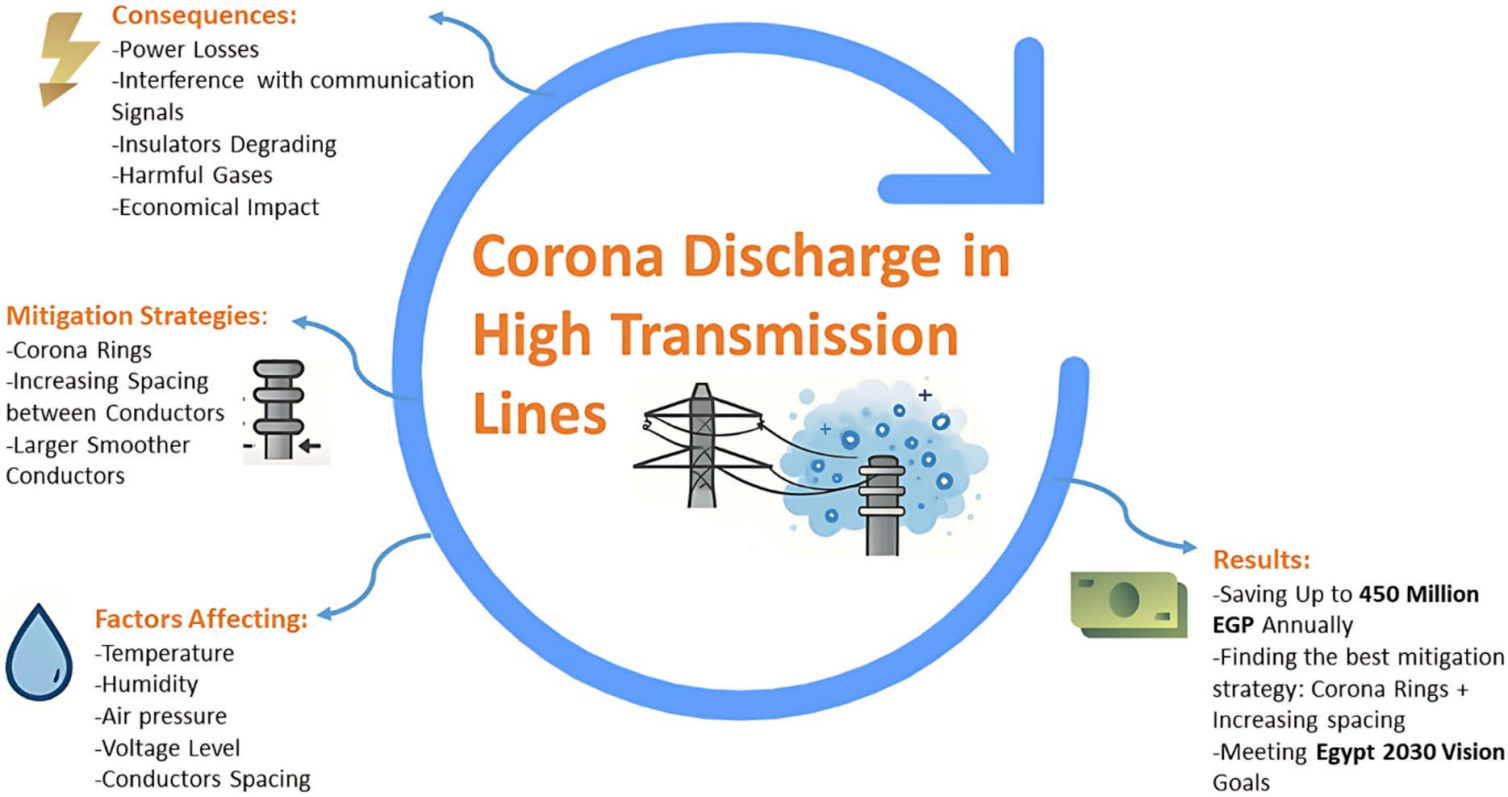
Graphical abstract.

Figure 2 summarizes the overall concept of the study. Starting from the factors affecting corona discharge (temperature, humidity, air pressure, voltage level, and conductor spacing), the figure illustrates how these parameters influence the initiation and severity of corona activity in high-voltage transmission lines. The resulting consequences include power losses, interference with communication signals, insulator degradation, release of harmful gases, and broader economic impacts. To address these issues, different mitigation strategies are highlighted, such as the use of corona rings, increasing spacing between conductors, and employing larger or smoother conductors. These interventions were selected for statistical evaluation in our simulations and analysis. Finally, the results section of the figure emphasizes the practical outcomes of applying the mitigation measures, including potential savings of up to 450 million EGP annually, identification of the most effective strategies (corona rings combined with increased spacing), and alignment with national energy efficiency targets such as Egypt’s 2030 vision goals. The figure therefore functions as a graphical abstract that links the physical causes of corona discharge with its technical, environmental, and economic implications, while also pointing to the data-driven solutions proposed in this paper.

The paper is organized as follows: Section I introduces the background and motivation of the study. Section II (Methdology) presents the analytical framework used to investigate corona discharge, including the application of Peek’s Law, air density correction models, degradation rate equations, and statistical techniques such as regression analysis, ANOVA, independent t-tests, and Monte Carlo simulation. The simulation environment, based on MATLAB/Simulink, and the design of scenario-based economic evaluations are also described. The results and discussions are presented in Section III. The conclusions and future work are dicussed in Section IV.

## Methodology

This study used a combined theory, simulation, and statistics approach to understand corona discharge in high-voltage systems and how to reduce its effects. Although this study applies the framework to Egypt’s 220 kV transmission network, the modelling and statistical procedures are universally applicable and can be adapted to different national grids. The methodology focuses on answering six main statistical questions. Each one is supported by practical analysis, simulations, and simplified data to get clear results. Following data collection, a series of statistical techniques were applied to analyze the underlying relationships between variables and to forecast the economic impact of corona discharge. Methods such as regression analysis, correlation assessment, and probabilistic simulation were employed to quantify the influence of environmental and electrical parameters on corona losses^23^. Similar advanced modelling strategies have been successfully applied in other domains of renewable energy and power systems, such as fractional-order and stochastic differential approaches for wind turbine and photovoltaic modelling^24,25^. These studies reinforce the value of combining deterministic physics with probabilistic/statistical tools, which we adopt here for corona discharge analysis. These techniques facilitated the identification of the most significant contributing factors and supported the evaluation of mitigation strategies. The following subsection outlines the key statistical tests and models implemented in this study^26^.

**Fig. 3:**
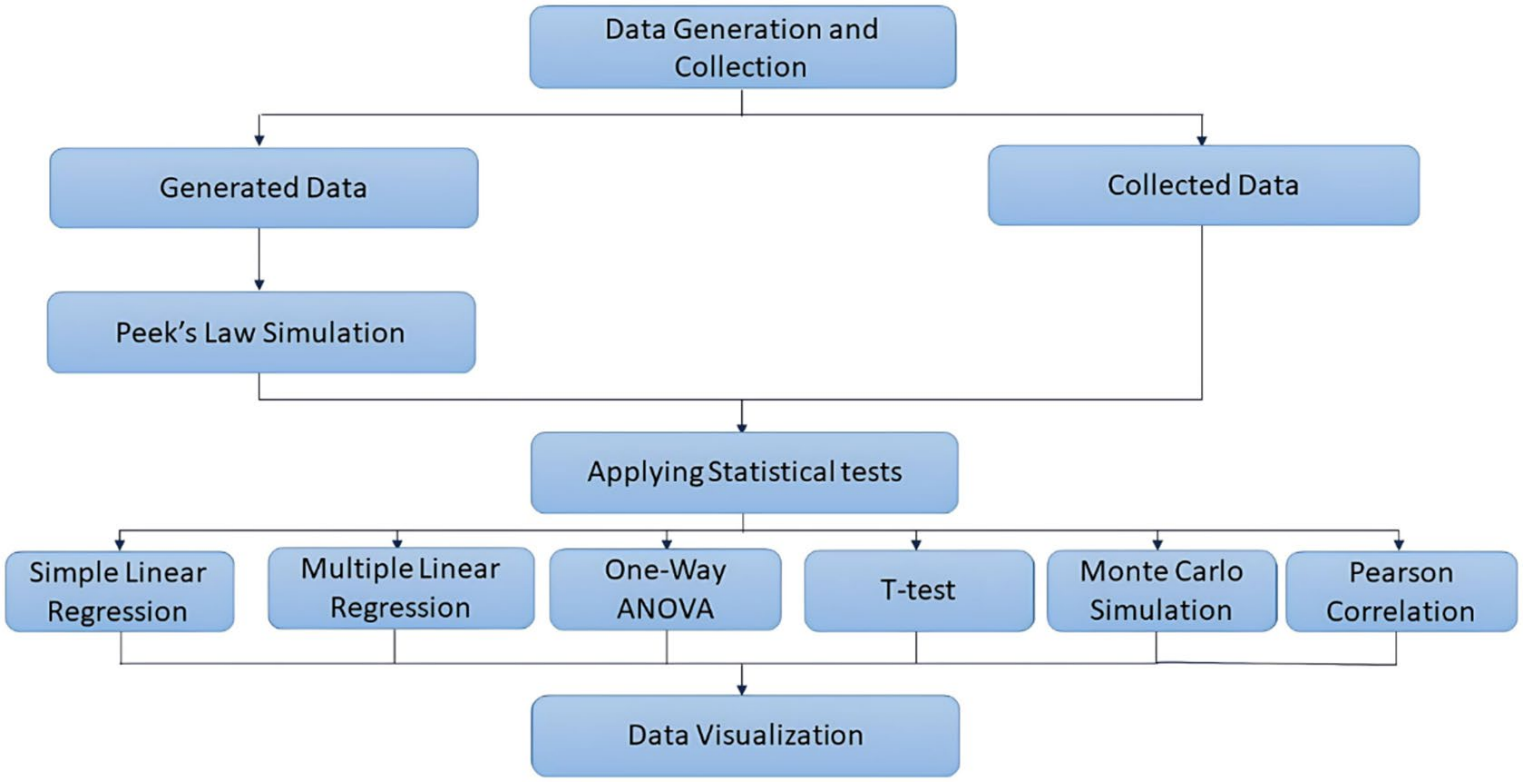
Overview of the data analysis workflow, including data preparation, statistical testing, and visualization.

To provide a clearer overview of the methodology, Fig. 3 illustrates the complete data analysis workflow adopted in this study. It begins with both the generation and collection of data, followed by the application of Peek’s Law simulation to the generated data. Subsequently, a range of statistical tests—including regression analyses, ANOVA, t-tests, Monte Carlo simulations, and Pearson correlation—were employed to extract meaningful insights and validate relationships among variables. The final stage involved visualizing the outcomes to support interpretation and communication of the results.

- Simple linear regression: Used to assess the linear relationship between two continuous variables by fitting a straight line that best represents the observed data^27^.
- Multiple linear regression: An extension of simple regression, this method was applied to model the relationship between a single dependent variable and multiple independent variables simultaneously^28^.
- One-way ANOVA: Utilized to determine whether statistically significant differences exist between the means of three or more independent groups^29^.
- Independent t-test: Conducted to compare the means of two independent groups and evaluate the significance of differences in their population means^30^.
- Monte Carlo simulation: A computational approach that leverages random sampling techniques to approximate the distribution of potential outcomes and support risk-based analysis^31^.
- Pearson correlation: Employed to quantify the strength and direction of linear association between pairs of continuous variables^32^.

### Application to Egypt’s 220 kV transmission system

To assess the economic impact of corona discharge on Egypt’s 220 kV transmission system, a Standard Monte Carlo Simulation (simple random sampling) was applied. The objective was to estimate the probabilistic distribution of annual corona-related energy losses and to evaluate the potential savings achievable through mitigation strategies.

####  Model Inputs

Deterministic parameters included the total line length of the 220 kV network (46,317 km)^33^, the annual operating hours (8,760 h/year), and the unit electricity tariff (1.6 EGP/kWh). The corona discharge loss rate was treated as the stochastic variable, modeled as a normal distribution with a mean of 1.5 W/m and a standard deviation of 0.2 W/m. This assumption is consistent with fair-weather conditions derived from Peek’s Law and validated through MATLAB/Simulink simulations and benchmark datasets (IEEE DataPort, Mendeley Data Repository).

#### Cost equation

For each iteration, the annual cost of corona discharge losses was calculated using the following equation:

1 $$\:C=H*T*\left(R*L\right)/1000$$

Where, *C* denotes the annual cost in Egyptian Pounds (EGP), *H* is the annual operating time (8,760 h), *T* is the electricity tariff (1.6 EGP/kWh), *R* represents the corona loss rate in watts per meter (treated as a random variable), and *L* is the total transmission line length in meters (46,317,000 m). The factor (÷ 1000) converts watts to kilowatts.

#### Simulation procedure

In each run, a random value of *R* was sampled from the normal distribution (mean = 1.5 W/m, standard deviation = 0.2 W/m) and substituted into the cost equation above to yield one realization of the annual loss cost. The procedure was repeated 10,000 times, generating a probabilistic distribution of outcomes. Convergence of the sample mean and variance was verified, confirming that 10,000 iterations were sufficient. No advanced variance-reduction techniques (e.g., Latin Hypercube or Sobol sequences) were required, as simple random sampling achieved stable results.

#### Hypothesis testing

To assess the likelihood of excessive losses, the following one-sided statistical hypothesis test was conducted:

- *H₀*: *P*(Cost > 900 million EGP) ≤ 0.5.
- *H₁*: *P*(Cost > 900 million EGP) > 0.5.

The null hypothesis was rejected, indicating that corona losses are more likely than not to exceed the 900 million EGP threshold.

#### Sensitivity analysis

In addition to the probabilistic framework, deterministic “what-if” scenarios were evaluated. By reducing the assumed average corona loss rate from 1.5 W/m to 1.0 W/m and 0.8 W/m, corresponding annual costs and expected savings were calculated. These scenarios provide a practical measure of the financial benefits achievable through feasible engineering interventions such as corona rings, increased conductor spacing, or surface treatments.

These analytical tools facilitated robust, data-driven insights and played a central role in validating the study’s findings.

In this study, an integrated analytical framework was developed to evaluate the behavior and impact of corona discharge phenomena under varying environmental and electrical conditions. The approach combined physical modelling, computational simulation, and statistical analysis to ensure a comprehensive understanding of the mechanisms involved. Physical modelling incorporated Peek’s Law, electric field threshold calculations, air density corrections, and degradation rate equations to establish the theoretical foundation for discharge prediction^34^. Simulated environmental data—specifically temperature, humidity, and pressure—were generated to replicate realistic atmospheric conditions affecting corona onset and intensity^35^. A MATLAB/Simulink-based model was implemented to simulate corona loss, accounting for interactions among voltage levels, conductor characteristics, and environmental factors. To assess economic implications, a scenario-based cost analysis was performed, evaluating different operational conditions and mitigation strategies. Finally, statistical methods were applied to quantify the relationships among key variables and to test the significance of observed effects, thereby reinforcing the reliability and applicability of the model. This multi-dimensional framework enabled a data-driven evaluation of corona discharge and supported informed decision-making for power system design and operation.

#### Research questions

1. Are there specific voltage thresholds beyond which the probability of corona discharge increases disproportionately?

The corona discharge threshold voltage for cylindrical conductors under standard atmospheric conditions is governed by Peek’s Law. This threshold depends on several factors, including conductor radius, spacing, surface condition, and air density. The onset voltage is estimated using the equation:

2 $$\:{e}_{v}={m}_{v}{g}_{v}rln\left(\frac{S}{r}\right)$$

where, $\:{e}_{v}$ is the visual corona onset voltage (kV), $\:{m}_{v}$ is the surface irregularity factor (typically 1.0 for smooth wires; 0.87–0.98 for rough or aged conductors), $\:r$ is the conductor radius (cm), $\:S$ is the distance between conductors, and $\:{g}_{v}$ is the visual critical electric field intensity (kV/cm).

The visual critical electric field $\:{g}_{v}$​ is further defined as:

3 $$\:{g}_{v}={g}_{0}\delta\:\left(1+\frac{c}{\sqrt{\delta\:r}}\right)$$

where, $\:{g}_{0}$ is the Disruptive critical electric field under standard conditions, $\:c$ is the empirical constant, and $\:\delta\:$ is the air density correction factor, calculated as:

4 $$\:\delta\:=\frac{P}{{P}_{0}}.\frac{{T}_{0}}{T}$$

with standard reference values: $\:{P}_{0}=76cmHg$(standard atmospheric pressure), $\:{T}_{0}=293K$ (standard temperature), and $\:P$ and $\:T$ are local pressure and temperature, respectively.

These expressions clearly demonstrate that the corona onset voltage is sensitive to several key parameters: conductor surface condition, spacing between conductors, and air density. Specifically, smoother conductors and greater inter-conductor spacing increase the voltage threshold required for corona initiation. Conversely, lower air density—often resulting from elevated temperatures or reduced atmospheric pressure—reduces this threshold. Accurate estimation of the onset voltage is therefore essential in the design of high-voltage systems to minimize energy losses and mitigate corona-related effects.

To validate the theoretical relationships and quantify the correlation between voltage and corona discharge behavior, a simple linear regression analysis was conducted on measured data comparing voltage levels to corona loss (Q1). The regression yielded a high coefficient of determination $\:{R}^{2}=0.921$, with a statistically significant *p*-value ($\:p<0.0001)$, confirming a strong linear relationship between voltage and corona-induced energy loss. This result empirically supports the theoretical predictions and underscores the importance of accurately modelling corona onset thresholds. Such modelling and statistical validation are crucial for minimizing energy losses and ensuring reliable design and operation of high-voltage transmission systems.

2. To achieve the objective of optimizing high-voltage system reliability, what is the correlation between corona discharge occurrences and system efficiency after adjusting for the influence of load variability?

System efficiency, the dependent variable, reflects how effectively the high-voltage (HV) or extra high voltage (EHV) system operates, capturing both energy losses and overall reliability. Corona discharge occurrences and load variability are treated as independent variables. Corona discharge arises from elevated surface electric field at the conductor, leading to energy dissipation, audible noise, and insulation aging. In HV/EHV networks, load variability influences corona indirectly via its impact on line voltage, not through low-voltage feeder imbalance. Specifically, light-load or open-circuit conditions can raise the receiving-end voltage through the Ferranti effect, while on-load tap-changer (OLTC)/AVR action and switching of shunt reactors/capacitors used for reactive-power control also shift operating voltage by a few percent. These HV mechanisms change the local field margin relative to the corona inception threshold: light load and reactive overvoltage tend to increase field stress and corona activity, whereas heavy load typically sags voltage and reduces corona severity. Consequently, managing load-driven voltage excursions with appropriate reactive-power compensation and voltage regulation is essential to minimize corona-related losses and maintain high system reliability.

To quantify these relationships, a multiple linear regression analysis was conducted (Q2), incorporating environmental and operational variables as predictors. The model yielded an $\:{R}^{2}$ value of 0.589, indicating a moderate but meaningful level of explanatory power. All predictors were found to be statistically significant$\:(p<0.0001)$, confirming their individual contributions to system efficiency. This statistical validation supports the hypothesis that both corona discharge and load variability are key determinants of performance in high-voltage systems. In practice, HV/EHV voltage excursions—principally Ferranti overvoltage at light load, reactive-power switching, and OLTC/AVR action—shift the surface-field margin relative to corona inception, thereby modulating corona activity. Therefore, efforts to control environmental fluctuations and mitigate discharge activity are crucial for improving efficiency and ensuring long-term system reliability.

3. What are the most significant positive applications of the corona discharge theory in high-voltage electrical power transmission systems? How can these applications improve system efficiency, reduce economic damage, and enhance environmental benefits?

Corona discharge, while typically regarded as a source of energy loss and insulation degradation, also offers several beneficial effects under certain conditions in high-voltage systems. One such benefit is voltage equalization, where the ionization of air around the conductor promotes a more uniform voltage distribution along extended transmission lines. This uniformity reduces the likelihood of excessive voltage differences developing at specific points, thereby enhancing line stability. Additionally, corona discharge contributes to surge protection during transient overvoltages caused by lightning strikes or switching operations. In such events, the discharge mechanism dissipates a portion of the excess energy, effectively acting as a buffer and mitigating the peak voltage stress experienced by the system. Furthermore, reduction of transient oscillations is another positive outcome. High-frequency oscillations resulting from sudden load changes or switching activities are dampened due to the inherent energy dissipation within the corona discharge process, which supports greater overall system stability. In addition to improving electrical system performance, this technology has been explored for unconventional uses—such as designing high-precision audio speakers—by utilizing the accurate pressure waves generated through corona-induced air movement.To evaluate and compare the effectiveness of various mitigation strategies aimed at harnessing or controlling corona discharge for these purposes, a one-way Analysis of Variance (ANOVA) was conducted (Q3). The statistical analysis yielded a highly significant result, with an F-value of 56.48 and a *p*-value of approximately 5.8 × 10^−17^, indicating strong differences among the strategies tested. This outcome validates the claim that not all mitigation approaches offer equal benefits and that certain methods are significantly more effective in optimizing the positive applications of corona discharge. Moreover, beyond conventional high-voltage engineering, corona discharge has demonstrated potential in non-electrical domains, such as precision audio technologies. Specifically, its ability to generate controlled acoustic pressure waves has been explored in the development of highly accurate speakers. Together, these findings suggest that controlled use of corona discharge can enhance system efficiency, reduce economic damage through improved reliability, and open new avenues for innovation in energy and acoustic applications.

## Results and discussion

### Statsitcal questions

1. To what extent does electrical voltage influence corona discharge power loss in high- voltage transmission lines, and is this relationship statistically significant?

To quantify the relationship between voltage and corona discharge loss in high-voltage systems, a simple linear regression analysis was conducted. In this model, corona discharge loss (kW) was treated as the dependent variable, while voltage (kV) served as the independent variable. The analysis revealed a statistically significant linear relationship between the two variables, with the regression equation expressed as:

5 $$\:\text{C}\text{o}\text{r}\text{o}\text{n}\text{a}\:\text{L}\text{o}\text{s}\text{s}=1.577\times\:Voltage-14.383$$

This model demonstrated a high degree of explanatory power, with $\:{R}^{2}=0.921$ and a *p*-value well below the 0.05 threshold $\:(p<0.0001)$, indicating a strong and statistically significant correlation between increasing voltage levels and elevated corona losses. These empirical findings are consistent with theoretical predictions based on Peek’s Law, which provides a physical basis for the onset and behavior of corona discharge in high-voltage environments. Peek’s formula relates corona power loss to parameters such as working voltage, conductor geometry, and frequency, as shown in the following expression:

6 $$\:{P}_{loss}=241\left(f+25\right)\left(\frac{\sqrt{r}}{S}\right){\left({E}_{n}-{E}_{v}\right)}^{2}\times\:{10}^{-5}$$

Where, $\:{E}_{n}$ is the working voltage, $\:{E}_{v}$​ is the corona generation voltage, $\:f$ is frequency, $\:r$ is the conductor-to-ground distance, and $\:S$ is the conductor diameter.

A formal hypothesis test on the regression slope $\:\left({\beta\:}_{1}\right)$further confirmed the statistical significance of this relationship. The null hypothesis $\:{H}_{0}:{\beta\:}_{1}=0$ (implying no voltage effect) was rejected in favor of the alternative $\:{H}_{1}:{\beta\:}_{1}\ne\:0$, supporting the conclusion that voltage is a significant predictor of corona discharge loss. These results highlight the critical role of voltage management in mitigating corona-related energy losses.

**Fig. 4:**
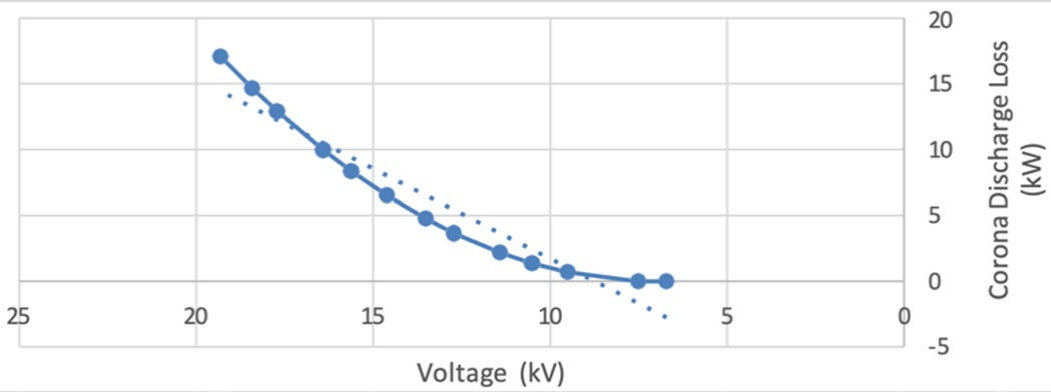
Scatter plot of corona discharge loss versus voltage, with the fitted linear regression line R^2^ = 0.921.

The findings are visually summarized in Fig. 4, which presents a scatter plot of corona discharge loss versus voltage, overlaid with the fitted linear regression line. The graph clearly illustrates the positive trend between increasing voltage and the fitted corona loss values, reinforcing the statistical interpretation of the data. This analysis underscores the importance of voltage control in maintaining efficient power transmission and minimizing energy dissipation due to corona effects.

Although the transmission corridor is designed for near-constant RMS voltage, operational HV effects (Ferranti at light load, reactive-device switching, and tap-changer action) introduce modest voltage excursions that shift the operating point relative to corona inception. Because corona loss rises rapidly once voltage exceeds CIV values, even small overvoltage during light-load periods can materially increase loss, while heavy-load voltage sag has the opposite effect.

2. How does the probability of corona discharge events change with varying environmental conditions (temperature, humidity, pressure)?

To further explore the factors influencing corona discharge loss in high-voltage systems, this study investigated the role of environmental conditions using a multiple linear regression model. The dependent variable was corona discharge loss (kW), while the independent variables included air temperature ($\:T$) in °C, relative humidity ($\:H$) %, and air pressure ($\:P$) in hPa. A dataset of 100 synchronized observations was compiled by combining Phasor Measurement Unit (PMU) records from the 220 kV transmission network with environmental data (temperature, humidity, and pressure) obtained from the nearest meteorological station. These observed data were chosen because they provide realistic operating conditions for evaluating corona discharge. While access to synchronized PMU–meteorological datasets may not be universally available, the methodology is not restricted to this dataset; comparable operational electrical and environmental data sources can be used in other contexts. To ensure reproducibility, we also referred to publicly available studies^27^ that validate the regression-based approach, confirming that the framework can be generalized beyond the specific dataset applied here. The synchronization of time stamps ensured that electrical and weather variables were aligned, enabling accurate regression analysis of corona discharge losses under real operating conditions. The regression equation derived from the model is as follows:

7 $$\:\text{C}\text{o}\text{r}\text{o}\text{n}\text{a}\:\text{L}\text{o}\text{s}\text{s}\left(\text{k}\text{W}\right)=0.054\times\:T+0.071\times\:H-0.033\times\:P$$

This model achieved an *R²* value of 0.589, meaning that nearly 59% of the variation in corona discharge loss could be explained by temperature, humidity, and pressure. This moderate level of explanatory power confirms that environmental factors significantly influence corona discharge, though additional unmeasured variables (e.g., pollution levels, wind speed) may also play a role (see Table 1). Hypothesis testing confirmed that all three environmental predictors had statistically significant effects on corona discharge loss $\:(p<0.0001)$. Specifically, temperature and humidity were found to increase corona loss, while air pressure exhibited a significant negative relationship. This negative correlation is consistent with Paschen’s law, which describes the dependence of breakdown voltage on the product of gas pressure and electrode spacing. As air pressure increases, the density of neutral molecules also increases, raising the CIV and making ionization less likely. In effect, higher pressure requires a stronger electric field for corona initiation, thereby reducing corona losses. Conversely, at lower pressures, fewer collisions are needed for ionization, which lowers the inception threshold and increases losses. The observed trend in Fig. 6 therefore agrees with established gas discharge theory. The null hypothesis $\:({H}_{0}:\text{a}\text{l}\text{l}\:\beta\:=0)$was rejected in favor of the alternative$\:({H}_{1}:\text{a}\text{t}\:\text{l}\text{e}\text{a}\text{s}\text{t}\:\text{o}\text{n}\text{e}\:\beta\:\ne\:0\:)$, confirming that environmental conditions exert a measurable impact on corona behavior.

**Fig. 5:**
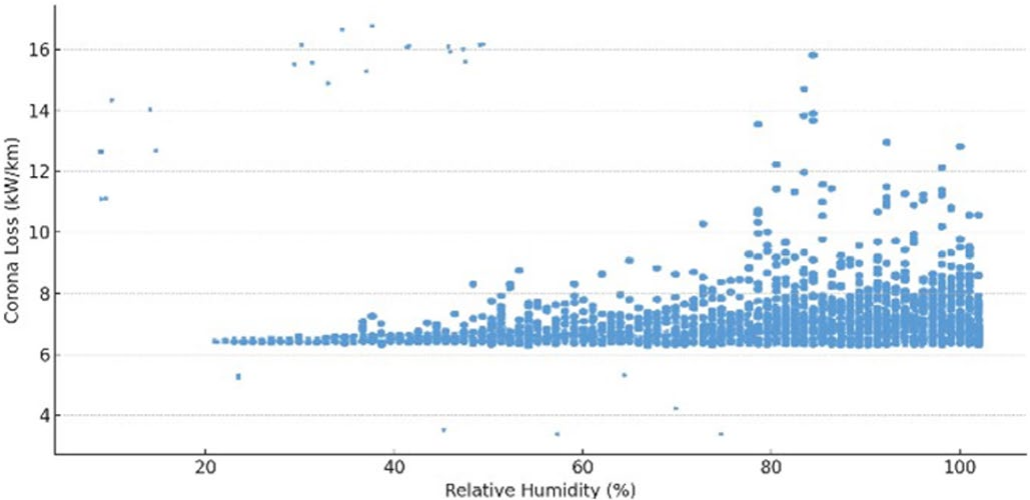
Scatter plot of corona discharge loss versus relative humidity, with an upward trend indicating a significant positive relationship (*p*-value $\:<\:0.0001$).

**Fig. 6:**
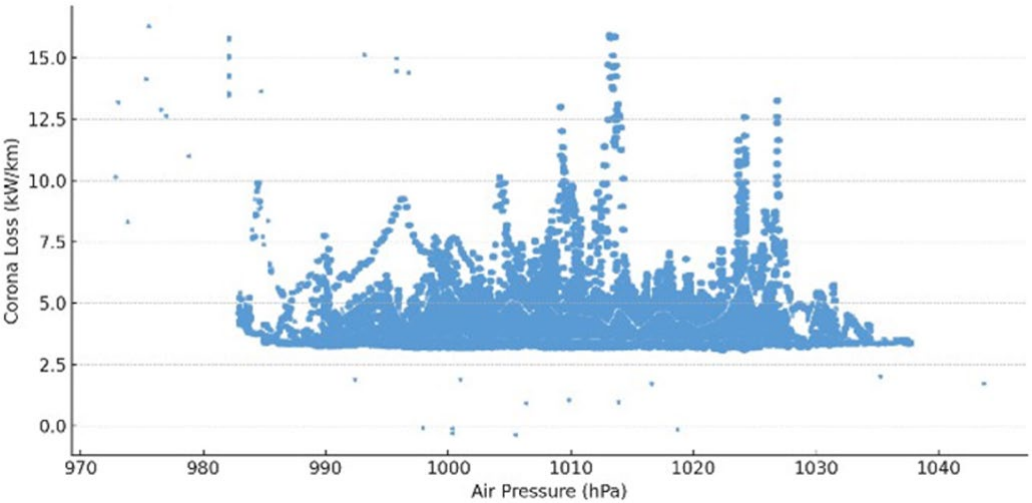
Scatter plot of corona loss versus air pressure showing a negative correlation, supporting the regression findings.

**Fig. 7:**
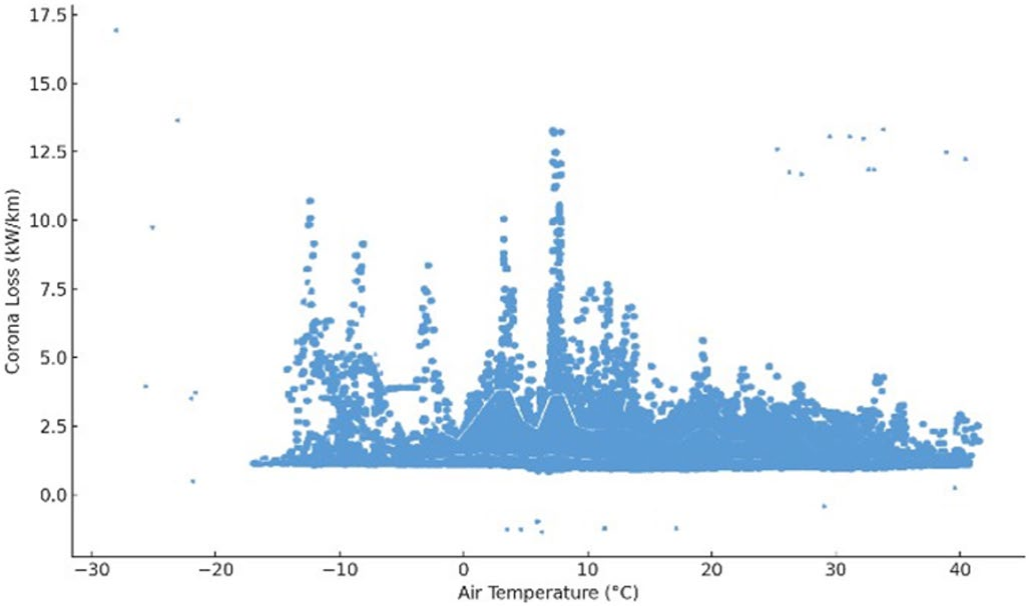
Corona loss versus air temperature, with a visible increasing trend and strong statistical significance.

These trends are visually supported by the scatter plots presented in Figs. 5, 6 and 7. Figure 5 illustrates a positive linear trend between corona discharge loss and relative humidity, reinforcing the regression’s statistical outcome. Similarly, Fig. 7 shows that increasing air temperature correlates with higher corona losses. In contrast, Fig. 6 demonstrates a negative correlation between air pressure and corona loss, consistent with the model’s prediction. Collectively, these results confirm that variations in environmental conditions—especially in temperature and humidity—significantly influence corona discharge characteristics. This finding is also consistent with the theoretical air density correction factor $\:\left(\delta\:\right)$, which accounts for pressure and temperature effects on corona onset behavior:

8 $$\:\delta\:=\frac{3.92\times\:P}{273+T}$$

This pressure trend is consistent with discharge physics. Paschen’s law states that inception depends on multiplication of gas pressure ($\:P$) and effective gap distance between electrodes ($\:d$) and has a minimum at small $\:P.d$; for air and sub-mm to a few mm effective distances near a conductor surface, our outdoor operating range lies to the right of that minimum, where inception increases with $\:P.d$.Therefore, within the observed barometric range (≈ 950–1020 hPa) and fixed geometry, higher pressure raises the corona inception field/voltage, yielding lower corona loss at a fixed operating voltage—precisely the negative correlation in Fig. 6. This is also captured by Peek’s air-density correction $\:\delta\:\:\propto\:\:P/T$, which scales the inception field accordingly.

In conclusion, environmental factors should be carefully monitored and accounted for in high-voltage system design and operation. The strong statistical significance of all predictors emphasizes the importance of adaptive system controls to mitigate corona-related energy losses under varying atmospheric conditions.

**Table 1:** Multiple linear regression results for environmental predictors of Corona discharge loss.

Variable	Coefficient (*β*)	*p*-value	Effect
*T* (°C)	+ 0.054	< 0.0001	Significant positive relationship
*H* (%)	+ 0.071	< 0.0001	Significant positive relationship
*P* (hPa)	– 0.033	< 0.0001	Significant negative relationship
*R*^2^ (model fit)	0.589	−	Model explains 58.9% of variance

3. To what extent do different mitigation strategies (e.g., conductor size, shape, spacing, and use of corona rings) significantly affect corona discharge loss in high-voltage transmission lines?

To evaluate the effectiveness of various corona mitigation strategies in reducing energy losses in high-voltage systems, a one-way ANOVA test was conducted. The independent variable was the mitigation strategy, categorized into five distinct groups: *Standard*,* Larger Conductor*,* Smooth Shape*,* Corona Ring*, and *Increased Spacing*. The dependent variable was corona discharge loss (kW). Each strategy group consisted of 10 simulated measurements derived from engineering estimates and theoretical performance metrics. The objective was to determine whether any strategy led to a statistically significant reduction in mean corona loss compared to the others.

The one-way ANOVA test yielded an F-statistic of 56.48 (*p* < 0.00001), which indicates that the variance in corona losses between mitigation strategies (e.g., corona rings, increased spacing) is far greater than the variance within each strategy group. This confirms that mitigation design choices produce statistically significant differences in performance. This allows us to reject the null hypothesis $\:{H}_{0}:{\mu\:}_{1}={\mu\:}_{2}={\mu\:}_{3}={\mu\:}_{4}={\mu\:}_{5}$​, which assumes equal mean losses across all strategies, in favor of the alternative hypothesis that at least one strategy significantly differs in effectiveness. The mean losses and standard deviations for each group are summarized in Table 2, highlighting that *Corona Ring* and *Increased Spacing* strategies achieved the lowest mean losses (1.5 kW and 1.6 kW, respectively), compared to the baseline *Standard* configuration at 2.5 kW.

**Fig. 8:**
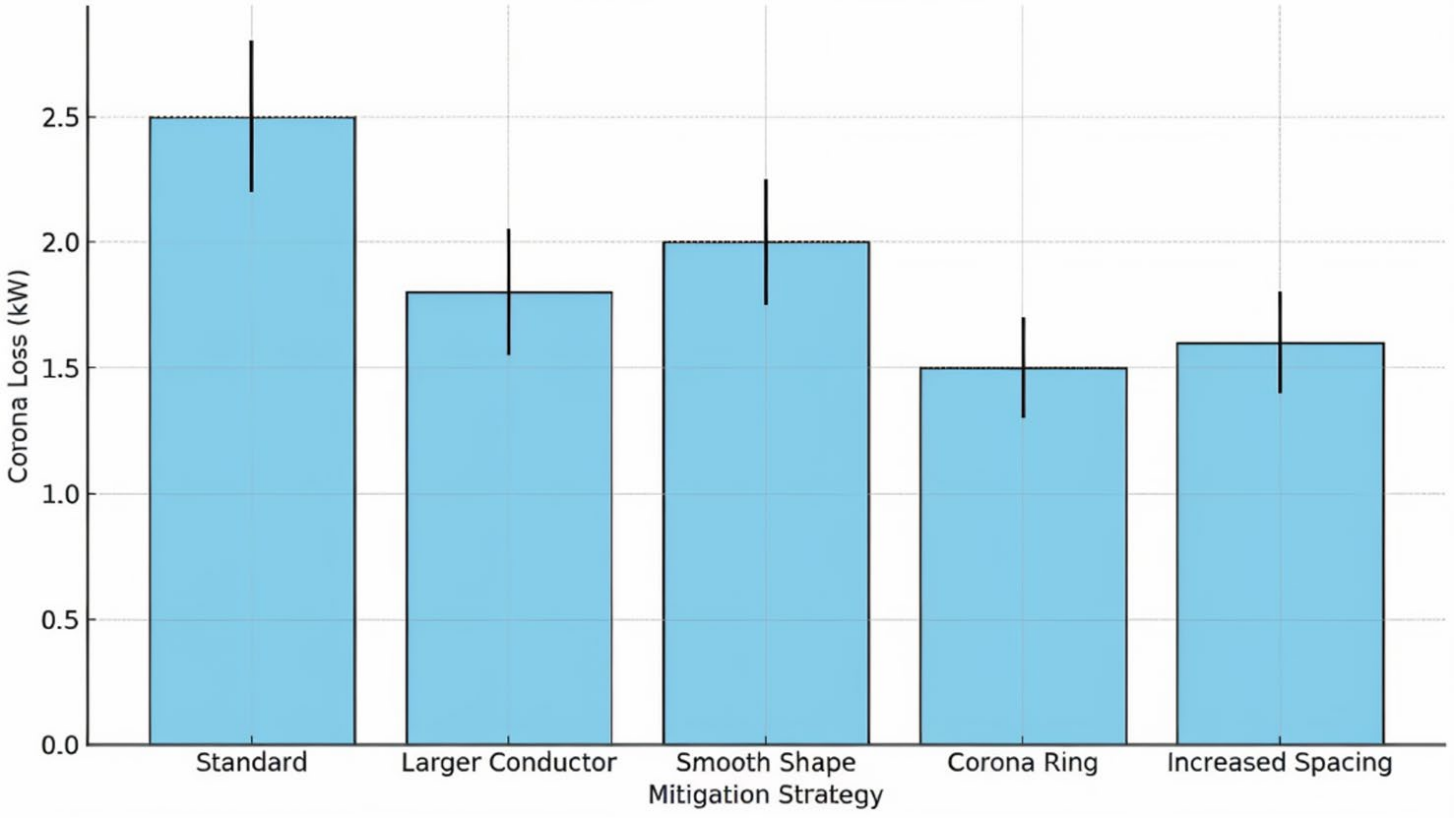
Average corona loss across mitigation strategies with standard deviation error bars. Simulated data used for illustrative comparison.

These findings are visually reinforced in Fig. 8, which presents a bar chart comparing the average corona discharge losses for each mitigation strategy, complete with error bars denoting standard deviation. The plot clearly shows that both the *Corona Ring* and *Increased Spacing* approaches outperform the others in terms of minimizing energy loss. This supports their practical implementation in high-voltage engineering to enhance transmission efficiency and reduce operational losses. Together, the statistical and visual analyses confirm the effectiveness of certain mitigation strategies in suppressing corona discharge and improving system reliability.

**Table 2:** Comparison of mean Corona discharge losses across mitigation strategies.

Strategy	Mean loss (kW)	Std Dev
Standard	2.5	0.3
Larger conductor	1.8	0.25
Smooth shape	2.0	0.25
Corona ring	1.5	0.2
Increased spacing	1.6	0.2

4. How does corona discharge affect the efficiency of radio systems across different frequency bands?

Corona discharge in Extra High Voltage (EHV) transmission lines is known to emit electromagnetic radiation that interferes with radio and television signals. This interference results in signal distortion or degradation, particularly in AM and Frequency Modulation (FM) broadcast bands. The severity of interference is influenced by several factors, including conductor design, conductor spacing, voltage level, and environmental conditions. Ensuring reliable communication and efficient power transmission requires a thorough understanding of these interference mechanisms, especially in areas prone to fluctuating atmospheric conditions or intense electromagnetic activity. A widely accepted model for estimating radio interference (RI) is expressed by the following empirical equation^36^:

9 $$\:RI=50+K\left({E}_{m}-16.95\right)+17.3686ln\left(\frac{d}{3.93}\right)+{F}_{n}+13.8949\text{ln}\left(20{D}_{1}\right)+F{F}_{w}$$

where, $\:{E}_{m}$ is the maximum electric field at the conductor surface, $\:d$ is conductor diameter, $\:{D}_{1}$​ is the lateral distance from the conductor to the antenna, $\:{F}_{n}$ is the number of conductors per bundle, and $\:F{F}_{w}$​ represents weather effects. The constant $\:K$ depends on the specific system configuration. This model quantifies the RI level in decibels, which can then be used to estimate television interference (TVI) in decibels (dB) using the following formula^37^:

10 $$\:TVI=RI-20{\text{log}}_{10}\left[\varphi\:\left(1+\left(Rh1\right)+\left(15h1\right)2\right)12\right]+3.2$$

Where, $\:RI$ is the base radio interference level resulting from corona activity, the variable $\:\varphi\:$ is a correction factor that accounts for receiver sensitivity and antenna directivity, $\:Rh1$ denotes the lateral distance between the receiving antenna and the nearest phase conductor, and $\:h1\:$is the height of the antenna above the ground, both measured in meters. The logarithmic adjustment models the attenuation of electromagnetic waves with distance and elevation, and the constant term (+ 3.2) serves as an empirical calibration offset based on standardized field measurements. This formulation provides a practical approach to estimating the potential impact of corona discharge on television signal quality, particularly in areas near high-voltage infrastructure.

To evaluate the impact of corona discharge across frequency bands, a two-sample t-test was performed comparing average interference levels in the AM band (0.5–1.6 MHz) and FM band (88–108 MHz). The results showed a statistically significant difference, with AM interference averaging 66.5 dB compared to 27.0 dB in FM. The *p*-value was approximately 3.60 × 10^−17^, and the effect size exceeded 14 standard deviations. This allowed rejection of the null hypothesis $\:{H}_{0}$​ (no difference between AM and FM interference), confirming that corona discharge disproportionately affects AM frequencies. Simulation results further demonstrated that AM readings consistently ranged from 64 to 69 dB, while FM ranged from 25 to 29 dB, with no overlap.

**Fig. 9:**
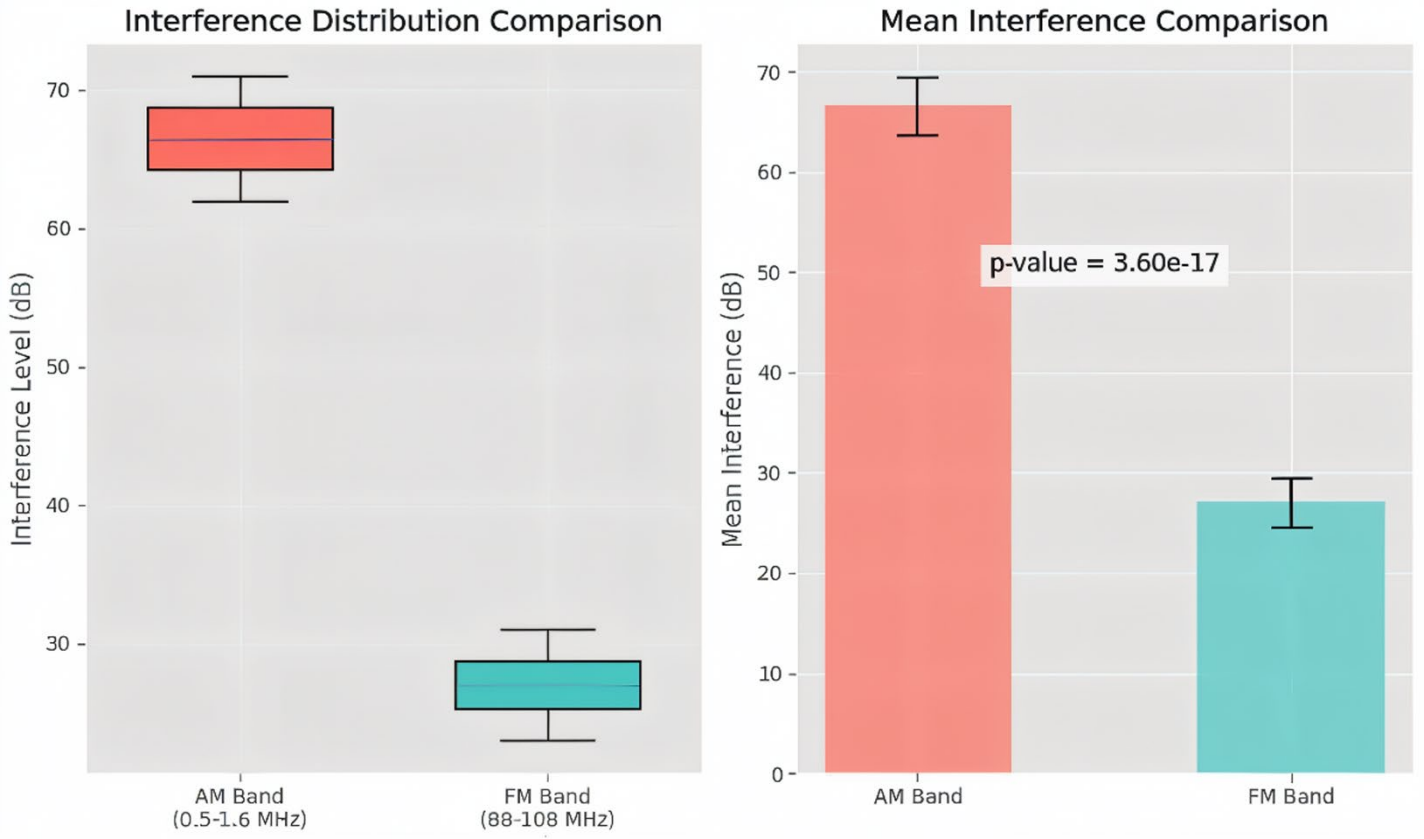
t-test simulation of Corona discharge-Induced radio frequency interference: Significant disparity in AM (0.5–1.6 MHz) versus FM (88–108 MHz) band susceptibility.

The disparity in interference levels is visually presented in Fig. 9, which illustrates both the distribution and mean comparison between AM and FM bands. The figure clearly highlights the higher susceptibility of AM bands to corona-induced disturbances, supporting the statistical conclusion. It should also be noted that conductor strand twisting (lay) can influence the spectral characteristics of corona noise. Twisting alters the effective distribution of surface electric fields and can cause partial cancellation of certain harmonics, thereby reducing interference at selected frequency bands. While this study did not explicitly quantify the effect of strand twisting, previous research has shown that it contributes to attenuation of higher-order harmonics and should be considered in detailed corona interference modelling^38^. These findings underscore the need for protective design measures to shield AM broadcast frequencies, particularly in high-voltage corridors. In contrast, FM systems remain relatively resilient, emphasizing their continued viability in electromagnetically noisy environments.

5. Does enhancing the design of electrical insulators (e.g., adding corona rings, using better materials) lead to a statistically significant reduction in degradation over time?

To assess the long-term effects of corona discharge on insulator performance, a comparative analysis was conducted between standard and enhanced insulator designs over a 10-year period. The dependent variable was the performance metric over time, measured in terms of degradation percentage, while the independent variable was the insulator design type. The study utilized a simulated dataset that captured the increasing effect of corona intensity on performance, with annual values recorded for both corona intensity (kV) and corresponding degradation %. These values, shown in Table 3, highlight the cumulative stress imposed by corona discharge and its impact on material durability.

A t-test was employed to compare the performance at year 10 between the two design types. The results indicated a statistically significant difference in degradation: the standard design exhibited a mean performance of 47.8%, whereas the enhanced design maintained a considerably higher performance of 83.6%. The *p*-value of approximately 8.7 × 10^−21^ confirms a highly significant distinction between the groups. Additionally, linear regression was applied to assess degradation trends over time, with low $\:{R}^{2}$ for both designs (0.005 for standard, 0.08 for enhanced), suggesting that degradation does not follow a strictly linear pattern, likely due to compounding effects of corona over time.

**Fig. 10:**
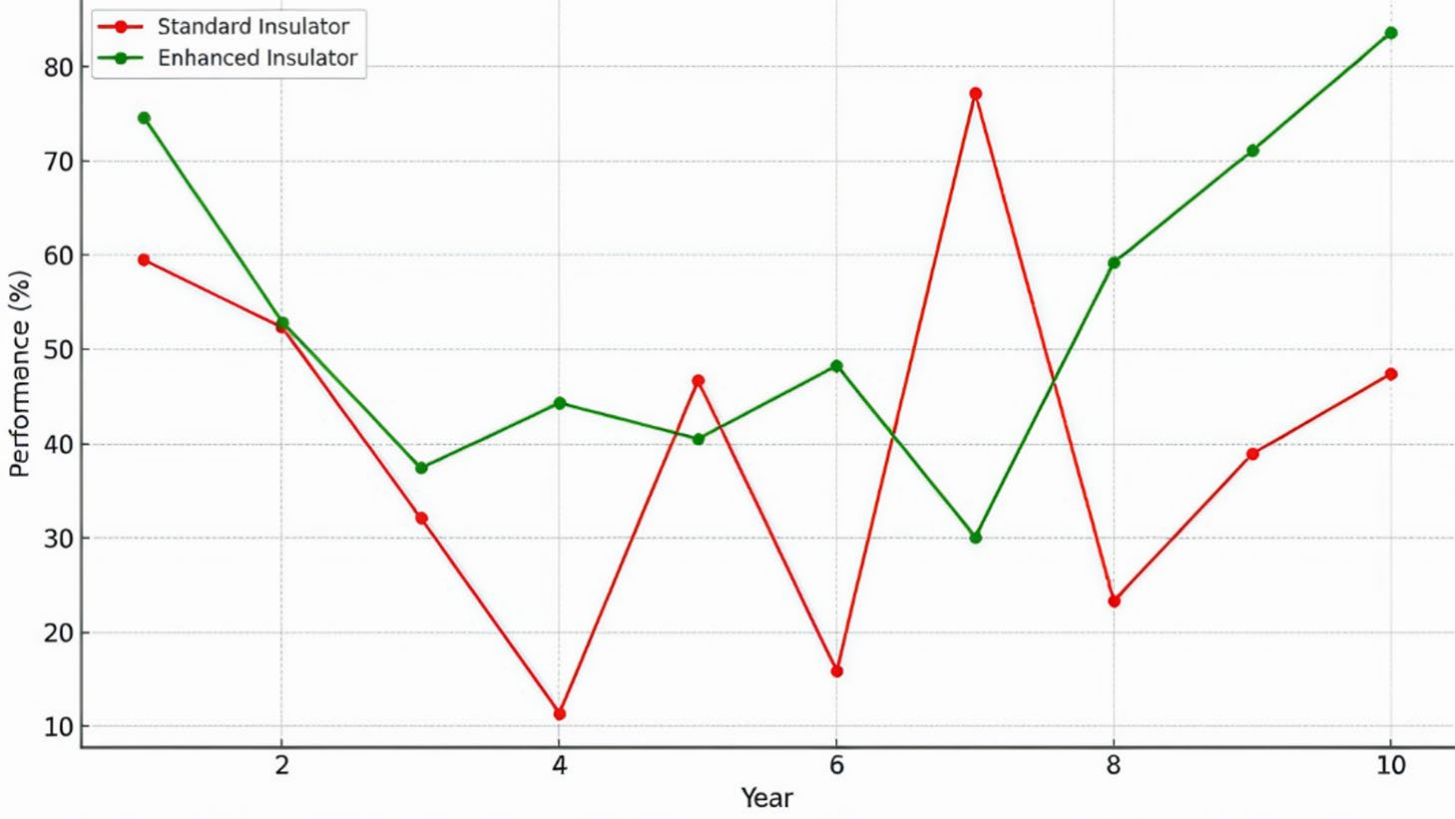
Degradation of standard vs. Enhanced insulators over 10 years.

These trends are further illustrated in Fig. 10, which plots the degradation trajectory of standard versus enhanced insulators across 10 years. The figure shows that enhanced insulators consistently outperform standard ones, especially in later years when corona intensity is highest. The observed difference supports the hypothesis that design improvements—such as material selection or structural enhancements—significantly improve insulator resilience under high-voltage corona stress. These findings advocate for the practical implementation of enhanced insulator technologies in modern high-voltage systems to ensure sustained reliability and reduced maintenance needs.

**Table 3:** Simulated Corona intensity and insulator degradation over 10 years.

Year	1	2	3	4	5	6	7	8	9	10
kV	7	9.6	12.1	14.7	17.2	19.8	22.3	24.9	27.4	30
%	24.6	28.2	37.8	43	48.4	53.6	59.8	68	71.2	83.6

6. On average, how much money (in EGP) can be saved annually by reducing corona discharge losses in high-voltage transmission lines?

To quantify the economic impact of corona discharge in high-voltage transmission systems, a Monte Carlo simulation was conducted to estimate annual energy loss costs under varying conditions. The analysis considered key independent variables, including transmission line length, corona loss rate (W/m), electricity tariff (EGP/kWh), and annual operating hours. The dependent variables were the estimated annual cost of corona-related losses and potential annual cost savings from mitigation strategies. As summarized in the model assumptions, a tariff rate of 1.6 EGP/kWh was used, with a total line length of 46,317 km, operating continuously for 8,760 h per year. The baseline corona loss rate was assumed to follow a normal distribution with a mean of 1.5 W/m and standard deviation of 0.2 W/m.

The statistical approach involved performing a Monte Carlo simulation with 10,000 iterations, each sampling the corona loss rate from the defined normal distribution. Using deterministic equations for cost estimation, the simulation produced a distribution of total annual loss costs. The hypothesis test evaluated whether the annual cost would exceed 900 million EGP in more than half of the simulated cases. The null hypothesis $\:{H}_{0}:P\left(\text{C}\text{o}\text{s}\text{t}>900M\:EGP\right)\le\:50\%$, was rejected in favor of the alternative $\:{H}_{1}:P\left(\text{C}\text{o}\text{s}\text{t}>900M\:EGP\right)>50\%$.

The Monte Carlo simulation produced the following outcomes:

- Mean annual cost: 973.5 million EGP.
- Standard deviation: 130.3 million EGP.
- Probability of cost exceeding 900 million EGP: 71.5%.

Deterministic sensitivity analysis revealed that reducing the corona loss rate from 1.5 W/m to 1.0 W/m could lower annual losses to approximately 648.2 million EGP, resulting in savings of 325.8 million EGP/year. A more aggressive reduction to 0.8 W/m would result in losses of approximately 519.2 million EGP, with savings of around 455 million EGP/year. Although these savings scenarios were calculated deterministically rather than simulated, they directly reflect the baseline model’s sensitivity and reinforce the economic case for proactive mitigation.

In conclusion, the Monte Carlo analysis confirms that annual corona discharge costs in Egypt’s 220 kV grid are highly variable but are likely to exceed 900 million EGP in over 70% of cases without mitigation. These results support the adoption of proactive strategies—such as conductor redesign, surface treatments, and insulation improvements—to reduce corona loss rates and improve economic performance.

**Fig. 11:**
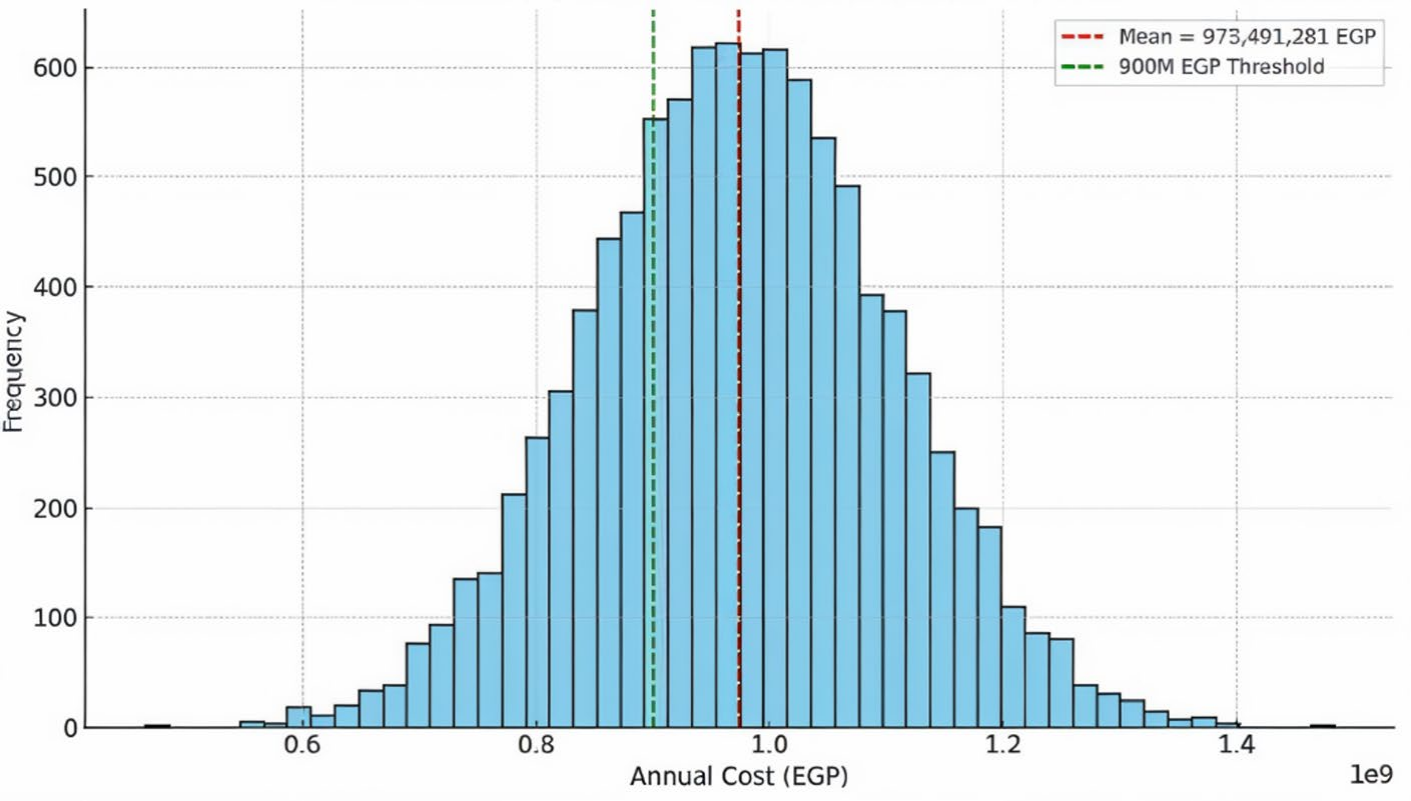
Monte Carlo simulation of annual corona loss cost showing mean and 900 M EGP threshold.

The simulation results indicated a mean annual cost of 973.5 million EGP, with a standard deviation of 130.3 million EGP. These findings are visually presented in Fig. 11, which displays the Monte Carlo distribution of annual loss costs, highlighting the 900 million EGP threshold and the concentration of outcomes around the mean. The figure illustrates the financial risk posed by uncontrolled corona discharge.

To explore cost-saving potential, deterministic calculations were used to estimate savings from mitigation strategies. Reducing the corona loss rate from 1.5 W/m to 1.0 W/m could lower annual losses to approximately 648.2 million EGP, yielding savings of 325.8 million EGP/year. A more aggressive reduction to 0.8 W/m would result in losses of about 519.2 million EGP, with savings approaching 455 million EGP/year. Although these savings were not independently simulated, they directly reflect baseline sensitivity to corona loss rates.

In conclusion, the Monte Carlo analysis confirms that annual corona discharge costs in Egypt’s 220 kV grid are highly variable but are likely to exceed 900 million EGP in over 70% of cases without mitigation. These results support the adoption of proactive strategies—such as conductor redesign, surface treatments, and insulation improvements—to reduce corona loss rates and improve economic performance.

Table 4 provides a summarized overview of the key statistical results from the study, highlighting the impact of electrical, environmental, design, and economic factors on corona discharge and system performance.

**Table 4:** Summary of statistical results across electrical, environmental, design, and economic variables.

Variable	Dependent variable	Statistical test	Result
Voltage (kV)	Corona loss (kW)	Linear regression	R^2^ = 0.921, *p*-value < 0.0001 (Higher voltage increases corona loss)
Temperature (°C)	Corona loss (kW)	Multiple linear regression	Coefficient: +0.054, *p*-value < 0.0001 (Higher temp = higher loss)
Humidity (%)	Corona loss (kW)	Multiple linear regression	Coefficient: +0.071, *p*-value < 0.0001 (Higher humidity = higher loss)
Pressure (hPa)	Corona loss (kW)	Multiple linear regression	Coefficient:-0.033, *p*-value < 0.0001 (Higher pressure = lower loss)
Mitigation strategies (Corona rings, spacing)	Corona loss (kW)	ANOVA	F-statistic = 56.48, *p*-value < 0.00001 (Corona rings and increased spacing significantly reduce losses)
Insulator design	Degradation rate (%)	T-test	*p* ≈ 8.7 × 10⁻²¹ (Enhanced insulators reduce degradation significantly over 10 years)
Economic analysis	Annual savings (EGP)	Monte Carlo simulation	Up to 455 million EGP savings with mitigation strategies

While Table 4 provides a summary of the statistical test results, Table 5 consolidates the overall outcomes of the study, linking each research focus area with the method applied, the key findings, and their practical implications.

**Table 5:** Summary of research outcomes.

Focus area	Method /test used	Key findings	Practical outcome
Voltage impact on corona loss	Simple linear Regression	Higher voltage significantly increases corona losses (*p* < 0.0001)	Emphasizes need for voltage management in HV lines
Environmental effects (T, H, P)	Multiple linear Regression	Temperature (+), Humidity (+) increase losses; Pressure (–) reduces losses (*R²* = 0.589)	Supports adaptive monitoring & control under changing weather
Mitigation strategies (conductor size, spacing, corona rings)	One-way ANOVA	Corona rings and increased spacing achieve lowest losses (*p* < 0.00001)	Confirms best engineering practices for HV design
Radio interference	Two-sample t-test	AM band heavily affected (≈ 66.5 dB), FM less affected (≈ 27 dB)	Need EMI-conscious infrastructure planning, protect AM frequencies
Insulator degradation	Independent t-test + trend analysis	Enhanced designs retain ~ 83.6% performance vs. 47.8% for standard	Justifies adoption of improved insulators
Economic analysis	Monte Carlo simulation	Annual corona-related loss ≈ 973.5 M EGP; mitigation saves up to 455 M EGP/year	Strong financial case for mitigation investments

This study demonstrates the significant influence of corona discharge on several key factors and the overall performance of high-voltage transmission networks. First, the analysis confirms that higher voltage levels significantly increase corona discharge losses, emphasizing the need for effective voltage management to minimize power loss and improve transmission efficiency. In terms of environmental influence, both temperature and humidity were found to elevate corona losses, whereas atmospheric pressure played a mitigating role. This underscores the importance of continuous environmental monitoring for accurate prediction and control of corona-related effects. With respect to mitigation, the study demonstrates that strategies such as corona rings and increased conductor spacing are particularly effective in reducing losses, reinforcing the necessity for thoughtful design enhancements in power systems.

**Fig. 12:**
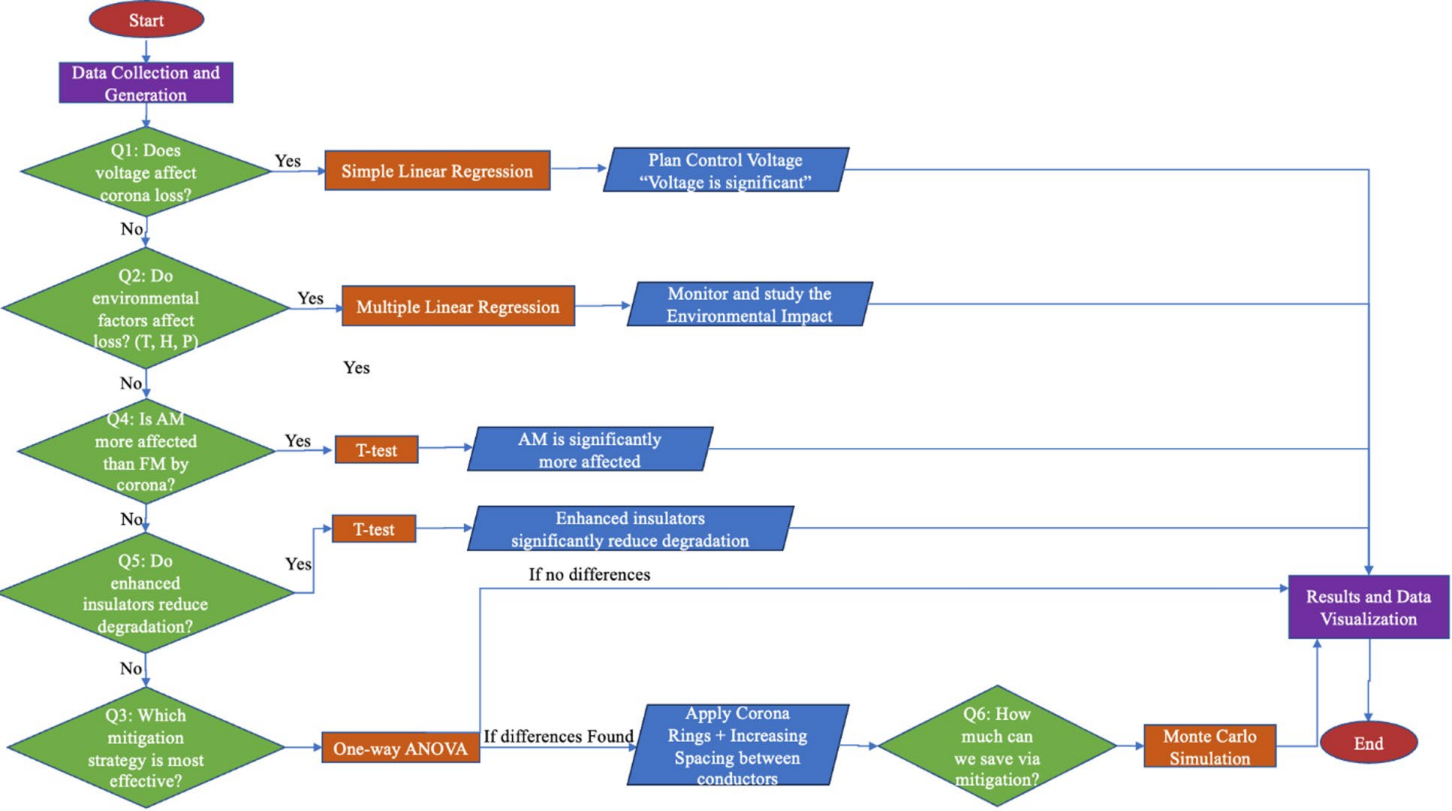
Flowchart of the statistical analysis and decision-making process for corona discharge evaluation.

Furthermore, the comparison of insulator types indicates that enhanced insulator designs significantly outperform standard ones in terms of degradation resistance under corona stress, supporting longer operational lifespans and improved system reliability. From an economic standpoint, the cost analysis revealed that implementing mitigation strategies could result in savings of up to 455 million EGP per year, clearly illustrating the financial benefits of addressing corona discharge proactively. To illustrate the logical sequence of the statistical analysis and decisions taken throughout this investigation, the methodology flowchart in Fig. 12 presents the structured progression from data collection to insight generation. Overall, the practical implications of these results highlight the value of integrating real-time environmental data and advanced material technologies to optimize the performance and cost-efficiency of modern transmission infrastructure.

## Conclusions and future work

This study comprehensively investigated the key factors contributing to corona discharge in high-voltage transmission systems by integrating theoretical modelling, simulation techniques, and statistical analysis. The research addressed critical questions surrounding the physical, environmental, and operational parameters that influence corona-related power losses and system efficiency. Findings demonstrated that both electrical and environmental variables significantly affect corona behavior. In particular, voltage exhibited a strong and statistically significant correlation with corona loss, emphasizing its critical role in system planning and regulation. Furthermore, temperature and humidity were shown to intensify corona discharge, whereas atmospheric pressure had a mitigating effect, consistent with Paschen’s law, since higher pressure raises the inception voltage and suppresses discharge losses—highlighting the value of environmental monitoring in predictive maintenance.

Design-based mitigation strategies, including the implementation of corona rings and increased conductor spacing, were validated as effective means to reduce corona losses. Additionally, enhanced insulator designs outperformed standard types in resisting long-term degradation, suggesting the importance of material and structural improvements for system longevity. From an economic standpoint, the study revealed that corona discharge could lead to substantial annual losses if left unmanaged; however, targeted mitigation could result in savings of up to 455 million EGP per year. Beyond energy efficiency, this research also highlighted significant electromagnetic interference, particularly in the AM radio band. Future work should consider the influence of conductor strand twisting, which may attenuate certain harmonics and alter the overall interference profile, underscoring the broader implications of corona discharge.

Overall, this research offers actionable, data-driven insights to improve the design, reliability, and economic performance of high-voltage transmission infrastructure. Future work should focus on the integration of real-time monitoring systems through SCADA platforms and advanced sensors to enable dynamic diagnostics and predictive control. Moreover, the application of machine learning models could improve forecasting of corona events and inform decision-making. Continued research on novel materials for conductors and insulators, alongside evaluation of long-term environmental and financial impacts, will be crucial in optimizing the next generation of resilient and efficient transmission systems. Although Egypt’s 220 kV grid was analyzed as the case study, the developed framework and findings are broadly generalizable and can be scaled to other national and regional high-voltage systems worldwide.

## Data Availability

The data that support the findings of this study are available from the corresponding author upon reasonable request.
